# Tricyclic Isatin Derivatives as Anti-Inflammatory Compounds with High Kinase Binding Affinity

**DOI:** 10.3390/molecules30142914

**Published:** 2025-07-10

**Authors:** Alexander V. Uvarov, Igor A. Schepetkin, Mark T. Quinn, Andrei I. Khlebnikov

**Affiliations:** 1 Kizhner Research Center, Tomsk Polytechnic University, Tomsk 634050, Russia; avu12@tpu.ru; 2 Department of Microbiology and Cell Biology, Montana State University, Bozeman, MT 59717, USA; mquinn@montana.edu

**Keywords:** dual-specificity tyrosine phosphorylation-regulated kinase 1 A (DYRK1A), proviral insertion site in Moloney murine leukemia virus (PIM), isatin, aryl oxime, NS-102, anti-inflammatory, cytokine

## Abstract

Oximes have been reported to exhibit useful pharmaceutical properties, including compounds with anticancer, anti-arthritis, antibacterial, and neuroprotective activities. Many oximes are kinase inhibitors and have been shown to inhibit various kinases. Herein, a panel of oxime derivatives of tricyclic isatins was synthesized and evaluated for inhibition of cellular inflammatory responses and binding affinity to several kinases. Compounds **5a** and **5d** (a.k.a. NS-102), which have an unsubstituted oxime group, inhibited lipopolysaccharide (LPS)-induced nuclear factor-κB/activating protein 1 (NF-κB/AP-1) transcriptional activity in human THP-1Blue monocytic cells and interleukin-6 (IL-6) production in human MonoMac-6 monocytic cells, with IC_50_ values in the micromolar range. These compounds also inhibited LPS-induced production of several other proinflammatory cytokines, including IL-1α, IL-1β, monocyte chemoattractant protein-1 (MCP-1), and tumor necrosis factor (TNF) in MonoMac-6 cells. Compounds **5a** and **5d** exhibited nanomolar/submicromolar binding affinity toward several kinase targets. The most potent inhibitor, **5d** (3-(hydroxyimino)-5-nitro-1,3,6,7,8,9-hexahydro-2*H*-benzo[*g*]indol-2-one), demonstrated high binding affinity for 12 kinases, including DYRK1A, DYRK1B, PIM1, Haspin, HIPK1-3, IRAK1, NEK10, and DAPK1-3. Molecular modeling suggested modes of binding interaction of selected compounds in the DYRK1A and PIM1 catalytic sites that agreed with the experimental binding data. Our results demonstrate that tricyclic isatin oximes could be potential candidates for developing anti-inflammatory drugs with neuroprotective effects for treating neurodegenerative diseases.

## 1. Introduction

Protein kinases play a central role in cellular signaling and regulation, serving as key modulators of various physiological processes. Dual-specificity tyrosine phosphorylation-regulated kinase 1 A (DYRK1A), proviral insertion site in Moloney murine leukemia virus (PIM), and Haspin (haploid germ cell-specific nuclear protein kinase) have emerged as significant kinase targets for the treatment of cancer, neurodegenerative diseases, and inflammatory diseases at the clinical and preclinical levels [1,2,3,4,5,6]. The known substrates of DYRK1A include proteins involved in transcription, cell cycle control, DNA repair, and other processes [7]. Overexpression of DYRK1A has also been proposed to be a significant contributor to the underlying neurodevelopmental abnormalities associated with Down syndrome [8]. Likewise, elevated DYRK1A immunoreactivity has been linked to neurofibrillary tangle pathology in Alzheimer’s disease (AD) [9]. Additionally, DYRK1A is involved in the phosphorylation of tau protein on sites that are hyperphosphorylated during the course of tau pathology in AD [1].

PIM proteins are evolutionarily conserved serine/threonine kinases consisting of PIM1-3. PIM1 is a constitutively active, highly conserved, pleiotropic, and multifunctional kinase that regulates many cellular functions, such as proliferation, apoptosis, and cell functional activity [2,3]. Recently, PIM1 inhibitors have been developed as potential therapeutics to reduce neuropathology in AD [10]. Haspin is highly upregulated in a variety of cancers, such as bladder cancer, colorectal, ovarian cancer, breast cancer, Burkitt’s lymphoma, and chronic lymphocytic leukemias [4,5,6]. Moreover, Haspin was expressed in the hippocampus and phosphorylated tau protein and may be one of the target molecules for the repression of tau phosphorylation in the treatment of AD [11].

Kinase inhibitors target several molecular pathways involved in differentiation and cellular growth and have been used in the treatment of cancer and inflammatory diseases [12]. For example, several pharmacological small-molecule inhibitors of PIM1 have been preclinically and clinically evaluated and were found to exhibit therapeutic potential [13,14]. DYRK1A inhibitors have a high therapeutic potential for pharmacological intervention in diabetes mellitus, neurodegenerative diseases, and cancer [15,16]. Although inhibitors of Haspin have not yet been widely explored, several scaffolds (e.g., derivatives of β-carboline, acridine, and 5-iodotubercidin) have emerged as agents with promising therapeutic potential [17].

Drug repositioning is the process of finding new clinical applications for existing drugs [18]. Off-target effects are typically unwanted and may be harmful, but in some cases, they have proven to be beneficial, resulting in new and unexpected uses for drugs [19]. Currently, heterocyclic compounds containing an oxime group in their structure are of considerable interest. For example, we found that 11*H*-indeno[1,2-*b*]quinoxalin-11-one oxime (compound IQ-1) and tryptanthrin-6-oxime derivatives inhibited enzymes of the c-Jun *N*-terminal kinase (JNK) family, which are regulators of inflammatory processes and cell proliferation [20,21,22,23]. Other aryl oximes with an isatin moiety have also been reported to have anticancer and anti-inflammatory potential [24,25]. It should be noted that the indole structure of isatin is a fundamental fragment of many known biologically active compounds of natural and synthetic origin [26]. Thus, it is reasonable that known aryl oximes could be used in screening of kinase targets to identify novel therapeutics. Additionally, multitargeting compounds to target multiple protein kinases as well as some receptors (i.e., *N*-methyl-d-aspartate (NMDA) receptors) involved in other processes related to AD could be useful for the development of potential therapeutics to treat neurodegenerative diseases [27,28].

There are several commercially available aryl oximes with known pharmacological effects. For example, it has been shown that domoic acid toxicity is reduced by treatment with the selective GluR6 antagonist NS-102 [29,30]. NS-102 was also used in other animal models as a GluK2 antagonist [31,32,33]. Thus, we pursued studies to provide further insight into the specificity of various previously described aryl oximes and identify novel and potential kinase inhibitors with anti-inflammatory activity. To accomplish this goal, NS-102 (3-(hydroxyimino)-5-nitro-1,3,6,7,8,9-hexahydro-2*H*-benzo[*g*]indol-2-one, designated in this report as compound **5d**) was screened across a panel of over 423 human kinases. Furthermore, several additional analogues of NS-102 (compounds **5a**–**c,e,f**) were synthesized and tested for biological activity in vitro. Importantly, molecular modeling suggested modes of binding interaction of compounds **5a** and **5d** in DYRK1A and PIM1 that agreed with the experimental binding data. We also evaluated activity of the structurally related oxime NS-383 (8-ethyl-6,7,8,9-tetrahydro-5-phenyl-1*H*-pyrrolo[3,2-*h*]isoquinolin-2,3-dione-3-oxime), which is an inhibitor of acid-sensing ion channels (ASICs) [18,34]. ASICs have also been linked to several neurological disorders, such as AD and Parkinson’s disease [35].

## 2. Results and Discussion

### 2.1. Chemical Synthesis

While certain tricyclic isatin analogues, including NS-102 and its precursors, have been previously reported [36], synthesis of these compounds in high purity has remained challenging due to the severe conditions required for their formation. In this study, we present optimized synthetic conditions for NS-102 (indicated here as **5d**) and its key precursors, 6,7,8,9-tetrahydro-1*H*-benzo[*g*]indol-2,3-dione and 5-nitro-6,7,8,9-tetrahydro-1*H*-benzo[*g*]indol-2,3-dione. Our approach aimed to achieve an optimal balance between preparative yield and purity of these compounds.

#### 2.1.1. Synthesis of NS-102 Precursors

**Scheme 1:**
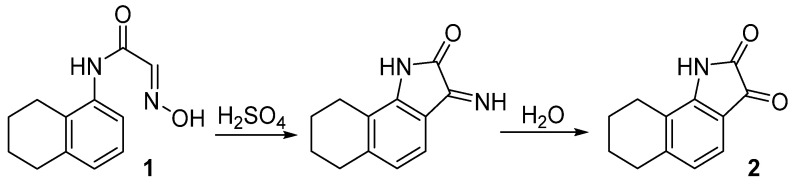
The synthesis of 6,7,8,9-tetrahydro-1*H*-benzo[*g*]indol-2,3-dione (**2**).

One of the methods for isatin ring formation utilizes Sandmeyer isatin synthesis [37], which uses unsubstituted oximes based on acetanilides. Sandmeyer intramolecular cyclization occurs in strongly acidic media and involves the *ortho*-carbon atom, resulting in formation of a fused pyrrole ring containing carbonyl and imino groups in positions *2* and *3*, respectively. The imino group of this intermediate can be readily hydrolyzed to form the second carbonyl group of the isatin. The Sandmeyer cyclization of 2-(hydroxyimino)-*N*-(5,6,7,8-tetrahydronaphthalen-1-yl)acetamide (**1**) to the NS-102 precursor 6,7,8,9-tetrahydro-1*H*-benzo[*g*]indol-2,3-dione (**2**) is represented in Scheme 1.

Despite the simplicity of the transformations during the synthesis of isatins according to Sandmeyer, some experimental difficulties arise due to structural features of acetanilides and tarring of the reaction mass in highly acidic media. The yield of isatins depends on the cyclization temperature (20–100 °C) and the concentration of sulfuric acid. In most cases, 98% sulfuric acid is used. Lower concentrations of H_2_SO_4_ can be used for the cyclization of easily sulfonated isonitrosoacetanilides; however, the resulting isatin may be contaminated with isatin-3-oxime [38]. Instead of H_2_SO_4_, some authors [39] have proposed the use of polyphosphoric acid (PPA) as a cyclizing agent. Nevertheless, in comparison with sulfuric acid, this method has not found wide application due to the possible formation of by-products, such as *N*-phenyloxamide derivatives [38].

Varying the concentration of sulfuric acid and temperature, we optimized conditions of the Sandmeyer synthesis to obtain the tricyclic isatin analogue **2** in good yields. The results showed that success of the cyclization depended strongly on the H_2_SO_4_ concentration. It was found that maintaining the temperature at 50 °C and using 75% solution of H_2_SO_4_ gave the highest yield of compound **2** (90%). In addition, the maximum level of acetanilide **1** conversion (98%) was achieved under these conditions. Our protocol minimized the formation of tarry by-products and led to compound **2** in sufficient purity for further synthetic applications without its chromatographic purification. Details of the optimization experiments are presented in Supplementary Materials Table S1.

**Scheme 2:**
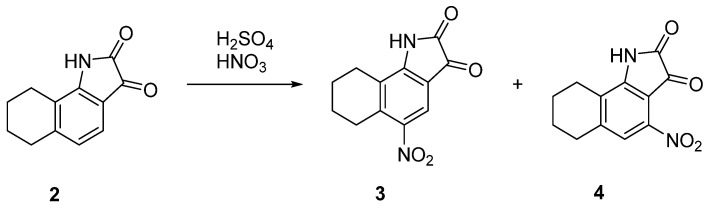
The nitration of 6,7,8,9-tetrahydro-1*H*-benzo[*g*]indol-2,3-dione.

One of the promising approaches for the functionalization of tricyclic isatin **2** is its nitration at the aromatic ring, which should be directed predominantly at position *5* of the heterocyclic system to form derivative **3** (Scheme 2).

Considering that the nitration of compound **2** can be achieved by the action of HNO_3_ in H_2_SO_4_, we tried to perform a one-pot synthesis of nitro derivative **3**. Isatin **2** was prepared according to Scheme 1, with subsequent addition of a small excess of concentrated nitric acid. We found that the 4-nitro-substituted isomer **4** was always present in the reaction products in a minor amount. Use of the previously described method [40] of isatin precipitation by the gradual addition of acid to a solution of water-soluble isatin salts to separate nitro derivatives **3** and **4** led to unsatisfactory results. Moreover, the yields of compounds **3** and **4** in the one-pot synthesis were low due to a significant tarring of the reaction mixture.

According to the NMR data, the nitration of compound **2** in concentrated H_2_SO_4_ (d = 1.84 g/cm^3^) with 1.2 eq. of HNO_3_ (d = 1.4 g/cm^3^) led to the formation of isomers **3** and **4** in a 9:1 ratio (Table 1) (see also [41]). The use of a 75% solution of H_2_SO_4_ in a mixture with 65% HNO_3_ at 5 °C almost eliminated the possibility of substitution at position *4* during the nitration of tricyclic isatin **2** (Table 1). Purification of the nitro derivative **3** was performed by dissolving the mixture in a 10% solution of NaOH and gradual precipitation with 1 M HCl.

#### 2.1.2. Oximation of Isatin Derivatives

**Scheme 3:**
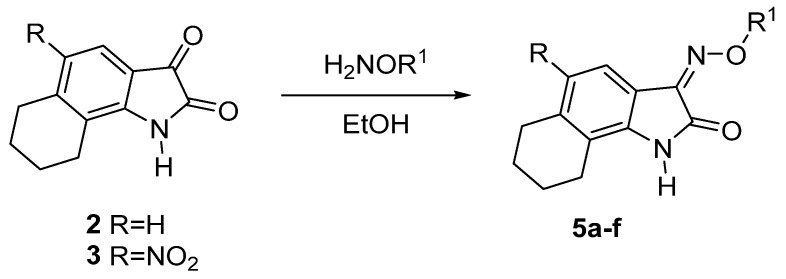
Oximation of the tricyclic isatins.

Considering that the oxime function is one of the most important pharmacophore groups, we obtained a number of oximes (Table 2) by the oximation of isatins **2** and **3** with hydroxylamine hydrochloride or *O*-substituted hydroxylamine hydrochlorides, according to Scheme 3. The reactions shown in Scheme 3 were performed at 78 °C using pyridine as a base, and the duration of the synthesis was 4 h. Oximes **5a**–**f** were obtained in 89–96% yields (Table 2). Due to the low solubility of isatin oximes, recrystallization can be carried out in a Soxhlet apparatus with methyl or ethyl alcohol. However, separation of the oxime precipitate from the reaction mixture and boiling it for 2–3 h in ethanol gave a product pure enough for further syntheses.

### 2.2. Biological Activity

#### 2.2.1. Anti-Inflammatory Activity (In Vitro)

Prior to evaluation of the compounds in cell-based assays, we measured their cytotoxicity in human monocytic THP-1Blue and MonoMac-6 cells during a 24 h incubation. NS-383 was cytotoxic in both cell lines. Thus, this compound was excluded from subsequent testing in cell cultures (Table 3).

**Figure 1:**
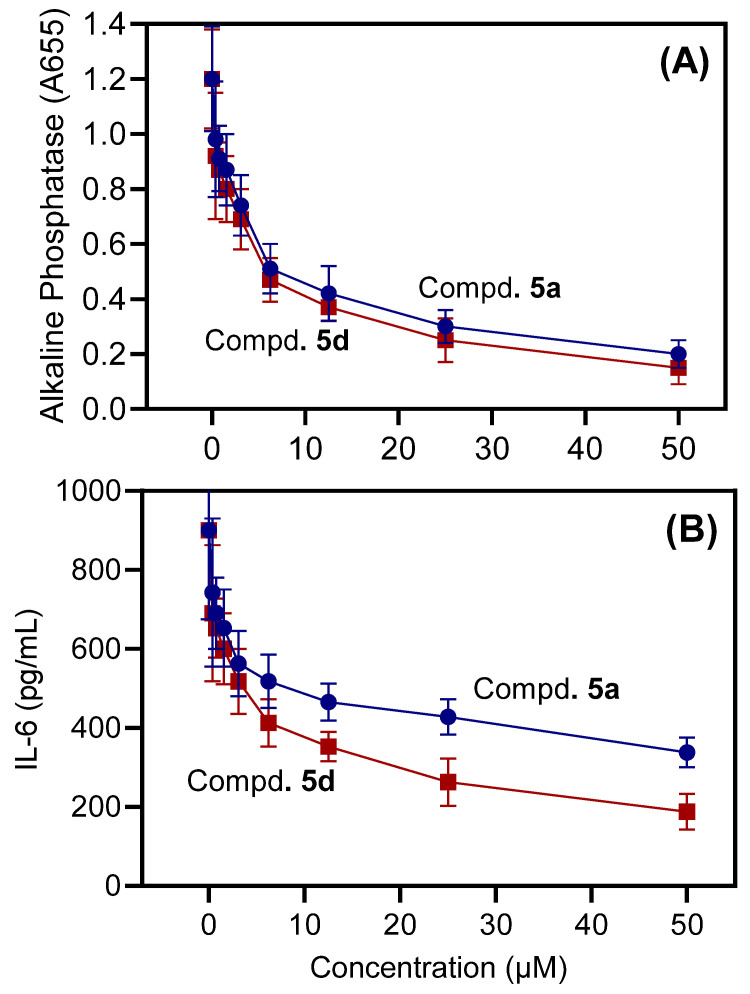
Analysis of the capacity of **5a** and **5d** to inhibit NF-κB/AP-1 activity and IL-6 production. (**A**): After THP-1Blue monocytic cells were treated with test compounds or control DMSO for 30 min, 250 ng/mL LPS or buffer was added, and the cells were incubated for 24 h. We analyzed NF-kB/AP-1 activity by measuring secreted alkaline phosphatase activity in the cell supernatants (absorbance at 655 nm). (**B**): After MonoMac-6 monocytic cells were treated with test compounds or control DMSO for 30 min, 250 ng/mL LPS or buffer was added, and the cells were incubated for 24 h. We determined IL-6 levels in the supernatants using ELISA, as described. The data in both panels represent the mean ± S.D. of triplicate samples from one experiment, which is from three independent experiments.

Compounds lacking cytotoxic activity were further evaluated for anti-inflammatory activity. Anti-inflammatory activity was assessed by determining the compound’s capacity to inhibit lipopolysaccharide (LPS)-induced NF-κB/AP-1 activity in THP-1Blue monocytic cells, as this pathway is essential in phagocyte inflammatory responses [42]. We also evaluated the effect of compounds on IL-6 production by MonoMac-6 monocytic cells. Compounds **5a** and **5d** were relatively potent inhibitors of NF-κB/AP-1 reporter activity in THP-1Blue cells, with IC_50_ values < 6.1 μM (Table 3). As an example, the dose-dependent inhibition of LPS-induced NF-κB/AP-1 activity by compounds **5a** and **5d** is shown in Figure 1A. Compounds **5a** and **5d** also inhibited IL-6 production in MonoMac-6 cells (Table 3). As an example, the dose-dependent inhibition of LPS-induced IL-6 production is shown in Figure 1B. These results indicate that alkylation of the oxime group (compounds **5b**, **5c**, **5e**, and **5f**) led to the loss of biological activity, indicating that the unsubstituted oxime function =N-OH is important for effective kinase binding.

**Figure 2:**
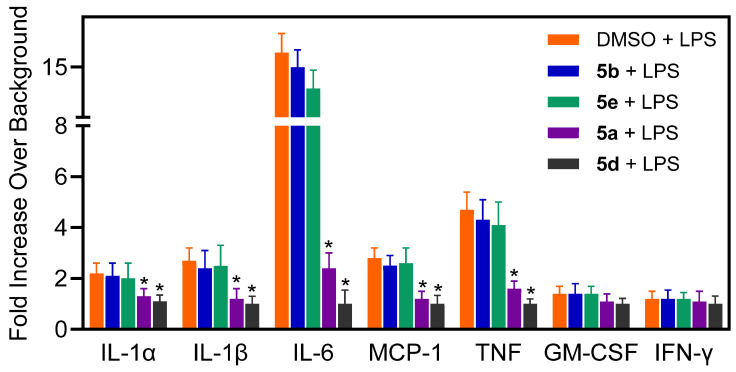
Effect of compounds **5a**, **5b**, **5d**, and **5e** on proinflammatory cytokine production in MonoMac-6 cells. Human MonoMac-6 monocytic cells were pretreated with 10 μM of the tested compounds or DMSO for 30 min, followed by addition of 250 ng/mL LPS or buffer for 24 h. Production of cytokines in the supernatants was evaluated using a Multiplex human cytokine ELISA kit. The relative level of cytokine production is shown as the fold increase over background (i.e., 1% DMSO control, without LPS). The data are presented as the mean ± S.D. of triplicate samples from one experiment that is representative of two independent experiments. * *p* < 0.05 in comparison with the control group (DMSO + LPS).

The effect of compounds **5a**, **5b**, **5d**, and **5e** on the production of an array of seven different proinflammatory cytokines and chemokines was also investigated using a Multiplex human cytokine ELISA kit. Compound **5d**, at a concentration of 10 μM, inhibited the secretion of IL-1α, IL-1β, IL-6, tumor necrosis factor (TNF), and monocyte chemoattractant protein-1 (MCP-1) in LPS-stimulated MonoMac-6 cells compared with dimethyl sulfoxide (DMSO)-treated control cells. The effect on granulocyte-macrophage colony-stimulating factor (GM-CSF) and interferon-γ (IFN-γ) production was inconclusive because of the low production of these cytokines after LPS treatment of MonoMac-6 cells (Figure 2). Compound **5a** was less effective in the inhibition of cytokine production, and compounds **5b** and **5e** were ineffective. These data further demonstrate the anti-inflammatory activity of **5a** and **5d**.

#### 2.2.2. Identification of Potential Kinase Targets

Protein kinases play critical roles in the regulation of NF-κB/AP-1 transcriptional activity. Thus, compound **5d**, which demonstrated potent inhibitory activity in all cell-based assays (Table 3), was profiled in a competition binding assay for its ability to compete with an active site-directed ligand for 423 different kinases (including mutated forms), representing all known kinase families (DiscoveRx KINOMEscan). Compound **5d** was screened at 10 μM, which was a concentration that effectively inhibited biological activity (see Figure 1). The kinases for which >96% inhibition of ligand binding and kinase activity was observed were designated as “kinase targets of the compound.” Twelve such kinase targets were identified, including DAPK1-3, DYRK1A, DYRK1B, Haspin, HIPK1-3, IRAK1, NEK10, and PIM1. Results for the percentage inhibition of binding to the active site-directed ligand for all of the 423 kinases after treatment with 10 μM **5d** are shown in Supplementary Materials Table S2, and data for the 12 selected kinase targets are shown in Table 4. The selectivity score, S(10), based on >90% inhibition of ligand binding at a single 10 μM screen concentration, was calculated by dividing the “kinase targets of the compound” with the “total number of nonmutated kinases” in the panel [43]. The published kinase profile of SP600125, a well-known c-Jun N-terminal kinase (JNK) inhibitor [44], was used to determine its S(10) value. We found that the S(10) for **5d** was much lower (0.073 = 31/423) compared with the S(10) for SP600125 (0.328 = 39/119), indicating much higher target kinase selectivity for **5d**. Three compounds (**5a**, **5d**, and NS-383) were submitted for determination of their binding affinities (K_d_) to the target kinases that exhibited the highest binding activity for compound **5d** in the primary kinase profiling. As shown in Table 4, compound **5d** had K_d_ values in the nanomolar range for all of tested kinase targets. Compound **5a** was less potent in the binding affinity, and NS-383 had low binding affinity for only PIM1.

Previously, we reported that aryl oximes with tryptanthrin and related scaffolds have a high binding affinity for JNK1-3 [20,21,22]. The isatin-type tricyclic oximes studied here exhibited relatively low binding affinity for JNK1-3 (52–76% at 10 µM concentration; Supplementary Materials Table S2). Although **5d** was inactive toward MKK7, this compound demonstrated a high binding activity (95.8% inhibition of the ligand binding) for mitogen-activated protein kinase 19 (MAP3K19), a kinase that directly activates the ERK and JNK cascades [45]. Thus, compound **5d** might be able to affect the JNK signaling pathway, although further studies are needed to assess this possibility.

### 2.3. Molecular Docking

**Figure 3:**
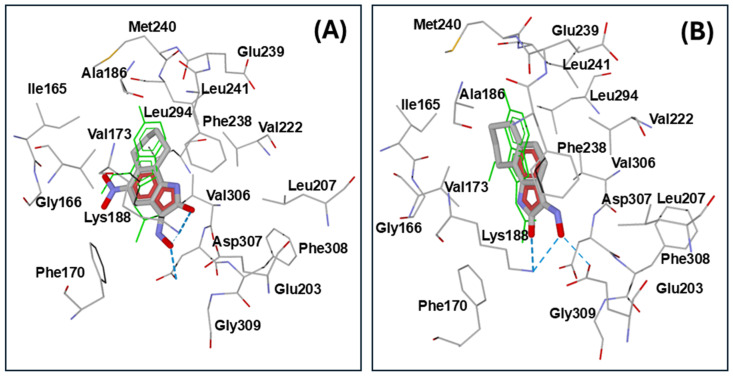
Docking poses of compounds **5d** (**A**) and **5a** (**B**) in DYRK1A binding site (PDB code 3ANQ). The co-crystallized ligand INDY [(1*Z*)-1-(3-ethyl-5-hydroxy-1,3-benzothiazol-2(3*H*)-ylidene)propan-2-one] is represented by thin green sticks. Residues within 4 Å from each pose are visible.

We performed molecular modeling of the potential interaction of compounds **5a** and **5d** with DYRK1A, as our kinase screening indicated high affinity of these compounds for DYRK1A. Using the known structure of DYRK1A (PDB code 3ANQ) [46], we found the best docking pose of **5d** presented in Figure 3A. Molecule **5d** was strongly anchored in the DYRK1A binding site by hydrogen bonding to Asp307 and Lys188, with participation of the oxime OH group and amide oxygen atoms. Analogously, compound **5a** formed H-bonds with Lys188, Glu203, and Asp307 of DYRK1A (Figure 3B, Table 5). Note that the amino acid residues in closest proximity to **5a** and **5d** were also located in the vicinity of co-crystallized DYRK1A/B inhibitor INDY [47]. The orientations of compounds **5a** and **5d** within the binding site are very similar, with the oxime and C=O groups directed towards Glu203 and Asp307. However, molecule **5a** is flipped with respect to **5d**, possibly because of steric bulkiness and polarity of the nitro group. The interface scores obtained for both isatin analogues with the ROSIE ligand docking protocol are nearly 14 units, with a slightly higher value for the nitro-derivative **5d**. The inactive ethyl-substituted oximes **5f** and NS-383, which was docked into DYRK1A for comparative purposes, had noticeably lower interface scores (Table 5). Although compound NS-383 is anchored by hydrogen bonding interactions within the binding sites of DYRK1A and PIM1 (Table 5), the presence of bulky phenyl group and ethyl substituent in the ligand may hinder its effective binding to these kinases.

**Figure 4:**
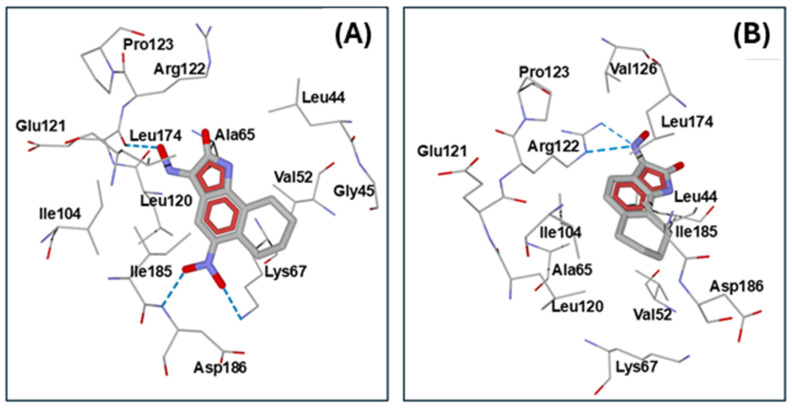
Docking poses of compounds **5d** (**A**) and **5a** (**B**) into the PIM1 binding site (PDB code 2OBJ). Residues within 4 Å from each pose are visible.

We also performed molecular docking of compounds **5a**, **5d**, and **5f** into another important kinase, PIM1 (PDB: 2OBJ). The nitro group of **5d** had strong hydrogen bonding interactions with Lys67 and Asp186 of PIM1, which may be responsible for different orientation of the molecule compared to **5a**. Additionally, the oxime OH group of **5d** formed a hydrogen bond with Glu121 (Figure 4A, Table 5). On the other hand, molecule **5a** had quite a different orientation in the binding site and was H-bonded to Arg122, with participation of the oxime nitrogen atom (Figure 4B). Additionally, the compounds **5a** and **5d** overlapped significantly with the position of the co-crystallized pyridone ligand (6-(5-bromo-2-hydroxyphenyl)-2-oxo-4-phenyl-1,2-dihydropyridin-3-carbonitrile) present in the PDB 2OBJ structure [48]. It should be noted that the nitro-derivative **5d** had a more negative interface score than compound **5a**, in accordance with a higher binding affinity to PIM1. The inactive *O*-Ethyl oxime **5f** and low-activity compound NS-383 (based on binding affinity toward PIM1, see Table 4) were characterized by a lower interface score (Table 5). These results, along with the data obtained for the docking of compound **5f** into DYRK1A (see above), highlight the importance of the unsubstituted oxime group =N-OH for effective binding to the investigated kinases.

NS-102 (i.e., compound **5d** here) has been reported to be a glutamate receptor and NMDA receptor antagonist and has been used in several in vivo and in vitro models of neurodegenerative diseases [29,30,31,32,33]. Thus, the effects of NS-102 in such experiments and in vivo models should be reevaluated to consider the potential anti-inflammatory and neuroprotective effects via inhibition of kinases, including DYRKA1, PIM1, and Haspin.

**Figure 5:**
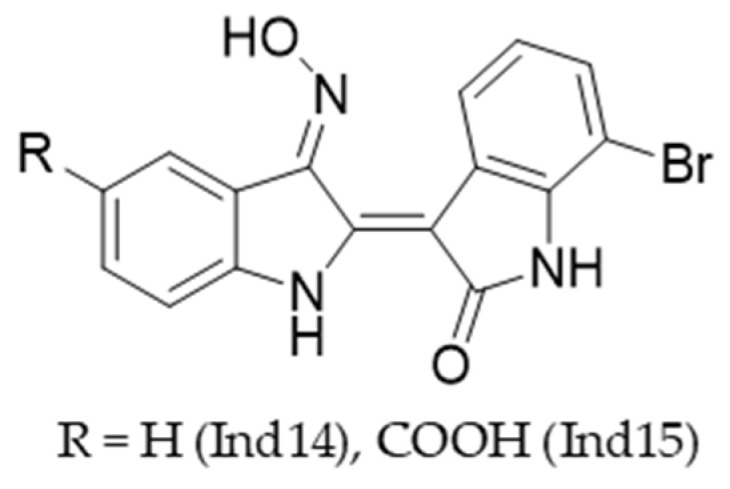
Chemical structures of indirubin oxime-based kinase inhibitors.

It should be noted that indirubin oximes Ind14 and Ind15 (Figure 5) were reported to demonstrate high affinity for various kinases, including DYRK1A potential [25]. Of these, the carboxylic acid derivative Ind15 acts as an inhibitor of both DYRK1A and DYRK2 [49]. Structurally, Ind14 and Ind15 contain indoline moieties and an unsubstituted oxime group, similar to isatins **5a** and **5d**. However, compounds **5a**–**f** are based on a structurally distinct tricyclic scaffold compared to the indirubin derivatives.

## 3. Materials and Methods

### 3.1. Chemistry

#### 3.1.1. Compounds and Reagents

NS-383 (8-ethyl-6,7,8,9-tetrahydro-5-phenyl-1*H*-pyrrolo[3,2-*h*]isoquinolin-2,3-dione-3-oxime) was purchased from Tocris Bioscience (Ellisville, MD, EUA). All the other starting reagents were purchased from Sigma-Aldrich (Burlington, MA, USA).

#### 3.1.2. Synthesis

Progress of the reactions was monitored by TLC on Sorbfil plates (sorbent—silica gel; base—aluminum; luminophore—254 nm). We determined the product melting points using a Stuart SMP30 instrument (Buch Holm, Herlev, Denmark). An Agilent Cary 630 FTIR (Agilent, Santa Clara, CA, USA) with an ATR attachment was used to obtain IR spectra. NMR spectra were recorded on a Bruker AM-400 spectrometer (Bruker, Billerica, MA, USA) with an operating frequency of 400 MHz (^1^H) and 100 MHz (^13^C). GC-MS analysis was performed on an Agilent 5975C instrument.

*6,7,8,9-Tetrahydro-1H-benzo[g]indol-2,3-dione* (**2**): 18 mL of 75% sulfuric acid (30 g) was added to a 50 mL round bottom flask. The solution was cooled in an ice bath, and 1 g of 2-(hydroxyimino)-*N*-(5,6,7,8-tetrahydronaphthalen-1-yl)acetamide (4.6 mmol) was added in small portions, avoiding strong heating of the reaction mixture. After the tetrahydronaphthalene derivative was completely dissolved, the flask was placed in a water bath heated to 50 °C. The reaction continued for 20 min (TLC monitoring, hexane-ethyl acetate, 2:1). The mixture from the reaction flask was poured onto ice, and the formed precipitate was filtered. Yield 88%. Orange crystals, M.p. 235–238 °C, IR (ν_max_, cm^−1^): 3439, 3188 (NH), 1730 (C=O), 1593, 1430 (C_Ar_-C_Ar_), 1199, 1060, 967, 834, 675. NMR ^1^H (DMSO-*d*_6_), δ, ppm: 10.92 (s, 1H, NH), 7.19 (d, *J* 7.8 Hz, 1H, ArH), 6.75 (d, *J* 7.8 Hz, 1H, ArH), 2.71 (t, *J* 6 Hz, 2H, CH_2_), 2.47 (t, *J* 6 Гц, 2H, CH_2_), 1.78–1.66 (m, 4H, CH_2_-CH_2_). NMR ^13^C (DMSO-*d*_6_), δ, ppm: 183.98, 160.58, 149.81, 149.29, 123.43, 121.43, 121.20, 115.00, 30.08, 23.07, 21.74, 21.56. GC/MS, *m*/*z*: 201.1 [M˙^+^].

*5-Nitro-6,7,8,9-tetrahydro-1H-benzo[g]indol-2,3-dione* (**3**): 10 mL of 85% sulfuric acid (17.8 g) was cooled in an ice bath, 1 g of compound **2** was added in portions, avoiding strong heating of the reaction mixture. In parallel, a nitrating mixture was prepared from 7 mL of 85% sulfuric acid (12.5 g) and 1 g of concentrated nitric acid 65% (2 eq. with respect to the substrate). The nitrating mixture was cooled to 5 °C and added dropwise to the solution of isatin **2** in sulfuric acid, maintaining the temperature in the range of 5–20 °C. Upon completion of the reaction (after 1 h), the contents of the flask were poured onto ice, and the resulting light-yellow precipitate was filtered and dried. Product yield 97%. Orange crystals, M.p. 253–254 °C, IR (ν_max_, cm^−1^): 3165 (NH), 1741 (C=O), 1614, 1593, 1525 (Ar-NO_2_), 1430 (C_Ar_-C_Ar_), 1355, 1302 (Ar-NO_2_). NMR ^1^H (DMSO-*d*_6_), δ, ppm: 11.47 (s, 1H, NH), 7.86 (s, 1H, ArH), 2.91 (t, *J* 6.5 Hz, 2H, CH_2_), 2.55 (t, *J* 6 Hz, 2H, CH_2_), 1.81–1.65 (m, 4H, CH_2_-CH_2_). NMR ^13^C (DMSO-*d*_6_), δ, ppm: 183.05, 161.02, 152.59, 145.03, 143.36, 123.34, 118.64, 114.95, 27.85, 24.13, 21.64, 20.75. LC/MS (ESI^+^); *m*/*z*: 269.0552 [M + Na]^+^ experimental ([C_12_H_10_N_2_O_4_ + Na]^+^ = 269.0538 theor.).

General procedure for the synthesis of oximes **5a**–**f**: In a 50 mL round bottom flask, 1 mmol of compound **2** or **3**, 1.2 mmol of a corresponding hydroxylamine hydrochloride, and 1.5 mmol of pyridine were dissolved into 20 mL of boiling ethanol. The reaction continued for 4 h. After complete conversion of the substrate (TLC monitoring), the reaction mass was poured into 100 mL of water, and the formed precipitate was filtered and dried. Product yields are given in Table 2.

*3-(Hydroxyimino)-1,3,6,7,8,9-hexahydro-2H-benzo[g]indol-2-one* (**5a**): Yellow crystals, M.p. 250–252 °C, IR (ν_max_, cm^−1^): 3165 (NH), 1693 (C=O), 1613 (C=N_oxime_), 1577, 1420 (C_Ar_-C_Ar_). NMR ^1^H (ДMCO-*d*_6_), δ, ppm: 13.06 (s, 1H, OH), 10.53 (s, 1H, NH), 7.65 (d, 1H, *J* 7.8 Hz, ArH), 6.72 (d, 1H, *J* 7.8 Hz, ArH), 2.69 (t, 2H, *J* 6 Hz, CH_2_), 2.53-2.45 (m, 2H, CH_2_), 1.78–1.63 (m, 4H, CH_2_-CH_2_). NMR ^13^C (DMSO-*d*_6_), δ, ppm: 165.90, 145.02, 142.07, 141.51, 124.38, 122.96, 119.74, 113.48, 22.93, 23.98, 22.57, 22.35. LC/MS (ESI^+^); m/z: 217.0977 [M + H]^+^ experimental ([C_12_H_12_N_2_O_2_ + H]^+^ = 217.0977 theor.).

*3-(Methoxyimino)-1,3,6,7,8,9-hexahydro-2H-benzo[g]indol-2-one* (**5b**): Yellow crystals, M.p. 237–239 °C, IR (ν_max_, cm^−1^): 3155 (NH), 1715 (C=O), 1602 (C=N_oxime_), 1586, 1430 (C_Ar_-C_Ar_). NMR ^1^H (DMSO-*d_6_*), δ, ppm: 10.65 (s, 1H, NH), 7.53 (d, 1H, *J* 7.8 Hz, ArH), 6.71 (d, 1H, *J* 7.8 Hz, ArH), 4.14 (s, 3H, CH_3_), 2.69 (t, 2H, *J* 6 Hz, CH_2_), 2.53–2.47 (m, 2H, CH_2_), 1.77–1.65 (m, 4H, CH_2_-CH_2_). NMR ^13^C (DMSO-*d*_6_), δ, ppm: 165.05, 144.74, 143.17, 142.22, 124.73, 123.11, 120.05, 113.10, 64.51, 29.99, 23.97, 22.47, 22.26. LC/MS (ESI^+^); *m*/*z*: 231.1135 [M + H]^+^ experimental ([C_13_H_14_N_2_O_2_ + H]^+^ = 231.1134 theor.).

*3-(Ethoxyimino)-1,3,6,7,8,9-hexahydro-2H-benzo[g]indol-2-one* (**5c**): Yellow crystals, M.p. 242–244°C, IR (ν_max_, cm^−1^): 3155 (NH), 1715 (C=O), 1601 (C=N_oxime_), 1430 (C_Ar_-C_Ar_). NMR ^1^H (DMSO-*d_6_*), δ, ppm: 10.65 (s, 1H, NH), 7.54 (d, 1H, *J* 7.8 Hz, ArH), 6.71 (d, 1H, *J* 7.84 Hz, ArH), 4.39 (q, 2H, *J* 7.08 Hz, CH_2_CH_3_), 2.68 (t, 2H, *J* 6 Hz, CH_2_), 2.53–2.46 (m, 2H, CH_2_), 1.76–1.65 (m, 4H, CH_2_-CH_2_), 1.71 (t, 3H, *J* 7 Hz, CH_3_). NMR ^13^C (DMSO-*d*_6_), δ, ppm: 165.18, 144.63, 143.00, 142.12, 124.64, 123.06, 119.97, 113.15, 72.28, 29.98, 23.97, 22.49, 22.27, 14.94. LC/MS (ESI^+^); *m*/*z*: 245.1295 [M + H]^+^ experimental ([C_14_H_16_N_2_O_2_ + H]^+^ = 245.1290 theor.).

*3-(Hydroxyimino)-5-nitro-1,3,6,7,8,9-hexahydro-2H-benzo[g]indol-2-one* (**5d**): Yellow crystals, M.p. > 255°C (decomp.), IR (ν_max_, cm^−1^): 3275 (NH), 1721 (C=O), 1616 (C=N_oxime_); 1504 (Ar-NO_2_); 1456 (C_Ar_-C_Ar_); 1300 (Ar-NO_2_). NMR ^1^H (DMSO-*d_6_*), δ, ppm: 13.73 (s, 1H, OH), 11.20 (s, 1H, NH), 8.32 (s, 1H, ArH), 2.93 (t, 2 H, *J* 6 Hz, CH_2_), 2.60 (t, 2H, *J* 6.5 Hz, CH_2_), 1.81-1.65 (m, 4H, CH_2_-CH_2_). NMR ^13^C (DMSO-*d_6_*), δ, ppm: 165.71, 145.47, 144.20, 143.59, 138.23, 121.53, 121.16, 112.76, 27.68, 24.64, 21.93, 20.98. LC/MS (ESI^+^); *m*/*z*: 262.0827 [M + H]^+^ experimental ([C_12_H_11_N_3_O_4_ + H]^+^ = 262.0828 theor.).

*3-(Methoxyimino)-5-nitro-1,3,6,7,8,9-hexahydro-2H-benzo[g]indol-2-one* (**5e**): Light-yellow crystals, M.p. > 264 °C (decomp.), IR (ν_max_, cm^−1^): 3308 (NH), 1748 (C=O), 1609 (C=N_oxime_), 1504 (Ar-NO_2_), 1450 (C_Ar_-C_Ar_), 1297 (Ar-NO_2_). NMR ^1^H (DMSO-*d_6_*), δ, ppm: 11.26 (s, 1H, NH), 8.16 (s, 1H, ArH), 4.23 (s, 3H, CH_3_), 2.9 (t, 2H, *J* 6 Hz, CH_2_), 2.56 (t, 2H, *J* 6.5 Hz, CH_2_), 1.78–1.67 (m, 4H, CH_2_-CH_2_). NMR ^13^C (DMSO-*d*_6_), δ, ppm: 166.50, 146.02, 143.88, 143.00, 140.13, 122.56, 120.15, 112.99, 60.32, 28.10, 24.55, 21.61, 20.06. LC/MS (ESI^+^); m/z: 276.0989 [M + H]^+^ experimental ([C_13_H_13_N_3_O_4_ + H]^+^ = 276.0984 theor.).

*3-(Ethoxyimino)-5-nitro-1,3,6,7,8,9-hexahydro-2H-benzo[g]indol-2-one* (**5f**): Light-yellow crystals, M.p. > 268 °C (decomp.), IR (ν_max_, cm^−1^): 3268 (NH), 1732 (C=O), 1603 (C=N_oxime_), 1520 (Ar-NO_2_), 1420 (C_Ar_-C_Ar_), 1383, 1323 (Ar-NO_2_). NMR ^1^H (DMSO-*d*_6_), δ, ppm: 11.26 (s, 1H, NH), 8.18 (s, 1H, ArH), 4.48 (q, 2H, *J* 7 Hz, CH_2_CH_3_), 2.9 (t, 2H, *J* 6 Hz, CH_2_), 2.57 (t, 2H, *J* 6 Hz, CH_2_), 1.78–1.65 (m, 4H, CH_2_-CH_2_), 1.37 (t, 3H, *J* 7 Hz, CH_3_). NMR ^13^C (DMSO-*d*_6_), δ, ppm: 167.70, 146.34, 144.16, 143.12, 139.81, 122.83, 120.01, 113.15, 74.70, 28.12, 24.63, 21.83, 19.99, 14.92. LC/MS (ESI^+^); m/z: 290.1151 [M + H]^+^ experimental ([C_14_H_15_N_3_O_4_ + H]^+^ = 290.1141 theor.).

### 3.2. Biological Assays

#### 3.2.1. Cell Culture

Human THP-1Blue monocytic cells stably transfected with a secreted embryonic alkaline phosphatase gene that is under control of a NF-κB/AP-1-inducible promoter were obtained from InvivoGen (San Diego, CA, USA). These cells were cultured in RPMI 1640 medium (Mediatech Inc., Herndon, VA, USA) containing 10% (*v*/*v*) fetal bovine serum (FBS), 100 μg/mL streptomycin, 100 U/mL penicillin, 100 μg/mL phleomycin (Zeocin), and 10 μg/mL blasticidin S (all from Sigma-Aldrich, St. Louis, MO, USA).

Human MonoMac-6 monocyte/macrophage cells (Deutsche Sammlung von Mikroorganismen und Zellkulturen GmbH, Braunschweig, Germany) were cultured in RPMI 1640 medium containing 10% (*v*/*v*) FBS, 10 μg/mL bovine insulin, 100 μg/mL streptomycin, and 100 U/mL penicillin.

#### 3.2.2. Evaluation of AP-1/NF-κB Activation

After incubation of THP-1Blue cells (2 × 10^5^ cells/well) with test compounds or control DMSO (1% final concentration) for 30 min, 250 ng/mL of lipopolysaccharide (LPS; from *Escherichia coli* strain 0111:B4) was added, and the cells were incubated for 24 h. Secreted alkaline phosphatase activity was assessed in the cell supernatants using the QUANTI-Blue mix (InvivoGen; absorbance at 655 nm) and compared with positive controls treated with LPS alone. Compound concentrations that inhibited 50% of the NF-κB reporter activity (IC50) were determined.

#### 3.2.3. Analysis of Cytokine Production

An IL-6 ELISA kit (BD Biosciences, San Jose, CA, USA) was used to assess the effect of selected compounds on IL-6 production. MonoMac-6 cells were plated in 96-well plates at a density of 2 × 10^5^ cells/well in culture medium supplemented with 3% (*v*/*v*) endotoxin-free FBS. Cells were pretreated with test compound or DMSO for 30 min, followed by addition of 200 ng/mL LPS for 24 h. For selected compounds, the IC_50_ for cytokine production was calculated by plotting percentage inhibition against the logarithm of inhibitor concentration (at least five points).

A multiplex human cytokine ELISA kit from Anogen (Mississauga, ON, Canada) was used to evaluate various cytokines (IL-1α, IL-1β, IL-6, GM-CSF, MCP-1, IFN-γ, and TNF) in the supernatants of MonoMac-6 cells.

#### 3.2.4. Cytotoxicity Assay

Cytotoxicity was analyzed with a CellTiter-Glo Luminescent Cell Viability Assay Kit from Promega (Madison, WI, USA) according to the manufacturer’s protocol. THP-1Blue cells were treated with varying concentrations of the test compounds (up to 50 µM) and cultivated for 24 h. After treatment, the cells were allowed to equilibrate to room temperature for 30 min, the substrate was added, and the samples were analyzed with a Fluoroscan Ascent FL (Thermo Fisher Scientific, Waltham, MA, USA).

#### 3.2.5. Kinase Profiling and K_d_ Determination

Kinase profiling was performed by KINOMEscan (Eurofins Pharma Discovery, San Diego, CA, USA), using a panel of 423 protein kinases, as described previously [43]. Compounds were submitted for dissociation constant (K_d_) determination toward several target kinases using KINOMEscan. In brief, kinases were produced and displayed on T7 phages or expressed in HEK-293 cells. Binding reactions were performed at room temperature for 1 h, and the fraction of kinase not bound to the test compound was determined by capture with an immobilized affinity ligand and quantified by quantitative polymerase chain reaction. The primary screening at fixed concentrations of the compound was performed in duplicate. For dissociation constant K_d_ determination, a 12-point half-log dilution series (a maximum concentration of 33 μM) was used. Assays were performed in duplicate, and their average mean values are displayed.

### 3.3. Molecular Docking

The 3D structures of ligands **5a** and **5d** were built using ChemOffice 2016 software, pre-optimized with the MM2 force field, and saved in the SDF format. The DYRK1A and PIM1 structures were downloaded from PDB (3ANQ [46] and 2OBJ [48] entries, respectively). These protein structures, along with prepared molecular models of the investigated isatin derivatives, were uploaded to the ROSIE web server [50], where the ligand docking protocol [51,52] was applied. The search areas for docking were positioned at the geometric centers of gravity of the (1*Z*)-1-(3-ethyl-5-hydroxy-1,3-benzothiazol-2(3*H*)-ylidene)propan-2-one and 6-(5-bromo-2-hydroxyphenyl)-2-oxo-4-phenyl-1,2-dihydropyridin-3-carbonitrile ligands co-crystallized in 3ANQ and 2OBJ structures, respectively. Up to 200 conformers were generated for each ligand with the use of the BCL conformer generation algorithm [53] incorporated in the ROSIE ligand docking protocol. For each of the compounds, 2000 docking runs were performed. The obtained docking poses with the most negative docking scores were saved and analyzed using the Molegro Virtual Docker 6.0 (MVD) program (CLC Bio, Copenhagen, Denmark).

## 4. Conclusions

Synthetic methods were developed to obtain tricyclic isatin analogue **2**, its nitro-derivative **3**, and the corresponding oximes **5**a and **5d**. These synthetic methods ensured high yields of the products along with good purity. The *O*-alkyl oximes **5b**, **5c**, **5e**, and **5f** were synthesized here for the first time and characterized. Evaluation of their biological activity showed that compounds **5a** and **5d** (a.k.a. NS-102) had potent anti-inflammatory activity and inhibited LPS-induced NF-κB/AP-1 transcriptional activity and proinflammatory cytokine/chemokine production by monocyte/macrophage cells. Analysis of their kinase targets showed that these compounds exhibited nanomolar/submicromolar binding affinity toward several kinase targets, including DYRK1A, DYRK1B, PIM1, Haspin, HIPK1-3, IRAK1, NEK10, and DAPK1-3. Furthermore, molecular modeling suggested modes of binding interaction of selected compounds in the DYRK1A and PIM1 catalytic sites that agreed with the experimental binding data. Thus, these tricyclic isatin oximes may have the potential to be developed into anti-inflammatory drugs, possibly for the treatment of neurodegenerative diseases.

## Figures and Tables

**Table 1:** The total yields and ratios of isomers **3** and **4** obtained on synthesis of 5-nitro-6,7,8,9-tetrahydro-1*H*-benzo[*g*]indol-2,3-dione.

Concentration of H_2_SO_4_ (%)	Total Yield of Isomers 3 and 4 (%)	Ratio of Products 3 and 4
98	10	9:1
75	89	20:1

**Table 2:** The oximation of isatin derivatives.

Compd.	R	R^1^	Yield (%)
**5a**	H	H	96
**5b**	H	Me	93
**5c**	H	Et	89
**5d**	NO_2_	H	95
**5e**	NO_2_	Me	90
**5f**	NO_2_	Et	90

**Table 3:** Analysis of compounds effects on LPS-induced NF-κB/AP-1 transcriptional activity, interleukin 6 (IL-6) production, and cytotoxicity.

Compd.	AP Production (THP-1 Cells)	IL-6 Production (MonoMac-6 Cells)	Cytotoxicity (THP-1 Cells)	Cytotoxicity (MonoMac-6 Cells)
	IC_50_ (µM)			
**5a**	6.0 ± 1.3	48.3 ± 4.4	N.T.	N.T.
**5b**	N.A.	N.A.	N.T.	N.T.
**5c**	N.A.	N.A.	N.T.	N.T.
**5d**	5.5 ± 1.4	12.6 ± 3.2	N.T.	N.T.
**5e**	N.A.	N.A.	N.T.	N.T.
**5f**	N.A.	N.A.	N.T.	N.T.
NS-383	n.d.	n.d.	19.2 ± 2.1	21.2 ± 2.4

N.T.: no cytotoxicity was found at concentrations up to 50 µM. AP: alkaline phosphatase. N.A.: compound was not active at the highest tested concentration (50 μM); n.d.: no data (activity of the compound was not evaluated because of its cytotoxicity).

**Table 4:** Inhibition of ligand binding by **5d** and K_d_ values for compounds **5d**, **5a**, and NS-383 against the twelve selected kinase targets.

Kinase Target	Inhibition (%) of Ligand Binding by 5d (10 µM)	Compd. 5d	Compd. 5a	NS-383
		K_d_ (µM)		
DAPK1	96.3	0.29 ± 0.02	13.5 ± 0.7	N.B.
DAPK2	98.9	0.20 ± 0.042	7.7 ± 1.1	N.B.
DAPK3	98.5	0.59 ± 0.028	20.5 ± 3.5	N.B.
DYRK1A	98.8	0.435 ± 0.078	0.85 ± 0.12	N.B.
DYRK1B	96.4	0.47 ± 0.02	N.B.	N.B.
Haspin	99.45	0.165 ± 0.021	0.61 ± 0.08	N.B.
HIPK1	98.5	0.52 ± 0.113	1.1 ± 0.07	N.B.
HIPK2	99.7	0.295 ± 0.092	0.73 ± 0.64	N.B.
HIPK3	100	0.135 ± 0.07	1.3 ± 0.3	N.B.
IRAK1	99.2	0.67 ± 0.113	N.B.	N.B.
NEK10	100	0.66 ± 0.071	3.2 ± 1.0	N.B.
PIM1	98.2	0.122 ± 0.054	1.8 ± 0.1	14.0 ± 0.7

N.B.: no binding was found at concentrations up to 30 µM.

**Table 5:** The pose characteristics obtained for the docking of compounds **5d**, **5a**, and **5f** into kinase targets DYRK1A and PIM1.

Kinase (PDB Code)	Compd.	Interface Score *	Hydrogen Bonds **
DYRK1A (3ANQ)	**5d**	−14.11	Lys188 (carbonyl oxygen, oxime oxygen-weak), Asp307 (oxime OH)
	**5a**	−13.97	Lys188 (carbonyl oxygen, oxime oxygen), Glu203 (oxime OH), Asp307 (oxime nitrogen-weak)
	**5f**	−13.50	Lys188 (NO_2_ oxygen), Asp307 (NO_2_ oxygen)
	NS-383	−13.19	Lys188 (oxime nitrogen), Asp307 (oxime OH), Gly309 (oxime OH-weak)
PIM1 (2OBJ)	**5d**	−12.74	Glu121 (oxime OH), Asp186 (NO_2_ oxygen), Lys67 (NO_2_ oxygen)
	**5a**	−12.24	Arg122 (oxime nitrogen)
	**5f**	−11.93	Lys67 (NO_2_ oxygen), Asp186 (oxime oxygen)
	NS-383	−11.39	Glu121 (oxime OH)

* Units of Rosetta docking score. ** Ligand groups involved in the formation of hydrogen bonds are shown in parentheses.

## Data Availability

Data is contained within the article and Supplementary Materials.

## Supplementary Materials

The following supporting information can be downloaded at: https://www.mdpi.com/article/10.3390/molecules30142914/s1, Table S1. Optimization of the reaction conditions on the synthesis of 6,7,8,9-tetrahydro-1*H*-benzo[*g*]indol-2,3-dione (compound **2**) from 2-(hydroxyimino)-N-(5,6,7,8-tetrahydronaphthalen-1-yl)-acetamide (compound **1**). Table S2. Kinase profile of NS-102 (compound **5d**). The kinases were evaluated using the KINOMEscan platform, as described under Materials and Methods. Shown is the percentage inhibition of binding to an active-site directed ligand for each of the indicated kinases after treatment with 10 μM NS-102.
