# Supplementary material for: Tricyclic Isatin Derivatives as Anti-Inflammatory Compounds with High Kinase Binding Affinity
Source: Molecules. 2025 Jul 10;30(14):2914. doi: 10.3390/molecules30142914 (PMC12300041; doi:10.3390/molecules30142914)
Supplement: Supplementary file 1 [file molecules-30-02914-s001.zip › molecules-3723036-supplementary.pdf]

Supplementary Materials

for

Tricyclic Isatin Derivatives as Anti-inflammatory Compounds with High Kinase Binding Affinity

Alexander V. Uvarov[1], Igor A. Schepetkin[2], Mark T. Quinn[2], and Andrei I. Khlebnikov[1]

[1] Kizhner Research Center, Tomsk Polytechnic University, Tomsk 634050, Russia and

[2] Department of Microbiology and Cell Biology, Montana State University, Bozeman 59717

Table S1. Optimization of the reaction conditions on the synthesis of 6,7,8,9-tetrahydro-1*H*-benzo[*g*]indole-2,3-dione (compound **2**) from 2-(hydroxyimino)-N-(5,6,7,8-tetrahydronaphthalen-1-yl)-acetamide (compound **1**).*

t, °C	98% H_2SO_4 **		75% H_2SO_4 ***		50% H_2SO_4 ****	
	Conversion of Compd. 1	Yield of Compd. 2	Conversion of Compd. 1	Yield of Compd. 2	Conversion of Compd. 1	Yield of Compd. 2
	%					
20	100	40	95	85	50	45
50	100	20	98	90	70	50
70	100	15	100	70	80	60
100	100	5	100	65	90	65

* Duration of the reactions in all the experiments was 20 min. Isolation and purification of isatin derivative **2** was performed by treating the reaction mixture with an aqueous 10% solution of NaOH (two-fold molar excess) to form a water-soluble sodium salt of compound **2**. Non-ionized organic by-products (tars, etc.) were removed by extraction with ethyl acetate, and isatin **2** was further precipitated by 1 M HCl from the aqueous layer. Another method used for purifying isatin **2** from resinous substances was to dissolve the crude product in isopropyl alcohol with the addition of excess NaOH dissolved in a minimum amount of water. The precipitate of isatin salt **2** was then filtered off and hydrolyzed with 1 M HCl.

** The reaction with concentrated 98% sulfuric acid at 0°C led to thickening of the reaction mass, and continuation of the synthesis became impossible. Increasing the temperature to 20°C or higher at the same H_2SO_4 concentration led to complete conversion of compound **1**. However, due to significant tarring, the yield of isatin **2** decreased with the increase in temperature. Thus, within the temperature range of 20 to 100°C, the use of 98% sulfuric acid was impractical due to the difficulties in separating the by-products formed.

*** Maintaining the temperature at 50°C and using 75% solution of sulfuric acid gave the highest yield of compound **2** (90%). Also, under these conditions, the maximum value of acetanilide **1** conversion (98%) was achieved.

**** The dilution of sulfuric acid to 50% was unsuitable for this transformation at 20°C, since a significant decrease in reactivity of the acetanilide derivative **1** was observed. Carrying out cyclization under these conditions over 48 h did not result in satisfactory results, since the product yield was about 50%. An increase in temperature led to a higher conversion of compound **1**, while the tarring of the reaction mixture also increased, which greatly complicated the isolation of the target product.

Table S2. Kinase profile of NS-102 (compound **5d**). The kinases were evaluated using the KINOMEscan platform, as described under Materials and Methods. Shown is the percentage inhibition of binding to an active-site directed ligand for each of the indicated kinases after treatment with 10 μM NS-102.

Gene Symbol	Inhib. (%)	Gene Symbol	Inhib. (%)	Gene Symbol	Inhib. (%)
AAK1	**91.8**	FAK	19	PCTK1	5
ABL1 (E255K)-P*	57	FER	39	PCTK2	0
ABL1 (F317I)-P	7	FES	0	PCTK3	0
ABL1 (F317I)-P	0	FGFR1	5	PDGFRA	0
ABL1 (F317L)-P	23	FGFR2	19	PDGFRB	0
ABL1 (F317L)-P	20	FGFR3	14	PDPK1	8
ABL1 (H396P)-non-P*	65	FGR	0	PFTAIRE2	6
ABL1 (H396P)-P	41	FLT1	12	PFTK1	0
ABL1 (M351T)-P	5	FLT3	12	PHKG1	0
ABL1 (Q252H)-non-P	55	FLT3 (D835H)	35	PHKG2	45
ABL1 (Q252H)-P	47	FLT3 (D835V)	72	PIK3C2G	43
ABL1 (T315I)-non-P	1	FLT3 (D835Y)	54	PIK3CA	0
ABL1 (T315I)-P	17	FLT3 (ITD)	29	PIK3CA (C420R)	0
ABL1 (Y253F)-P	41	FLT3 (ITD, D835V)	65	PIK3CA (E542K)	43
ABL1-non-P	54	FLT3 (ITD, F691L)	66	PIK3CA (E545A)	30
ABL1-P	46	FLT3 (K663Q)	5	PIK3CA (E545K)	0
ABL2	2	FLT3 (N841I)	0	PIK3CA (H1047Y)	36
ACVR1	2	FLT3 (R834Q)	10	PIK3CA (I800L)	5
ACVR1B	10	FLT3-autoinhibited	4	PIK3CA (Q546K)	40
ACVR2A	10	FLT4	8	PIK3CB	0
ACVR2B	25	FRK	0	PIK3CD	42
ACVRL1	0	FYN	0	PIK4CB	**95.9**
ADCK3	12	GAK	49	PIKFYVE	28
ADCK4	12	GRK1	67	PIM1	**98.2**
AKT1	10	GRK2	48	PIM2	**96**
AKT2	0	GRK3	77	PIM3	**95.8**
AKT3	9	GRK4	34	PIP5K1A	54
ALK	46	GRK7	43	PIP5K2B	64
ALK (C1156Y)	43	GSK3A	57	PIP5K2C	40
AMPK-α1	28	GSK3B	29	PKAC-α	0
ANKK1	8	HASPIN	**99.45**	PKAC-β	2
ARK5	24	HCK	20	PKMYT1	0
ASK1	50	HIPK1	**98.5**	PKN1	9
AURKB	**90.7**	HIPK2	**99.7**	PKN2	84
AURKC	39	HIPK3	**100**	PLK1	71
AXL	17	HIPK4	63	PLK2	67
BIKE	75	HUNK	0	PLK3	51
BLK	23	IGF1R	0	PRKCD	10
BMPR1A	18	IKK-α	52	PRKCE	61
BMPR1B	19	IKK-β	50	PRKCH	0
BMPR2	64	IKK-ε	23	PRKCI	49
BMX	13	INSR	1	PRKCQ	7
BRAF	28	INSRR	4	PRKD1	61

BRAF (V600E)	23	IRAK1	**99.2**	PRKD2	77
BRK	0	IRAK3	32	PRKD3	77
BRSK1	36	IRAK4	86	PRKG1	11
BRSK2	31	ITK	0	PRKG2	6
BTK	39	JAK1 (JH1domain-cat.)	0	PRKR	6
BUB1	**95.6**	JAK1 (JH2domain-pseudokinase)	89	PRKX	0
CAMK1	38	JAK2 (JH1domain-cat.)	42	PRP4	38
CAMK1B	12	JAK3 (JH1domain-cat.)	49	PYK2	0
CAMK1D	65	JNK1	76	QSK	14
CAMK1G	29	JNK2	49	RET	8
CAMK2A	71	JNK3	52	RET (M918T)	8
CAMK2B	70	KIT	0	RET (V804L)	2
CAMK2D	51	KIT (A829P)	0	RET (V804M)	4
CAMK2G	25	KIT (D816H)	1	RIOK2	80
CAMK4	7	KIT (D816V)	22	RIOK3	19
CAMKK1	9	KIT (V559D)	0	RIPK1	0
CAMKK2	56	KIT (V559D, T670I)	55	RIPK2	0
CASK	2	KIT (V559D, V654A)	0	RIPK4	55
CDC2L1	0	KIT-autoinhibited	0	RIPK5	17
CDC2L2	0	LATS1	0	ROCK1	61
CDC2L5	13	LATS2	28	ROCK2	54
CDK11	1	LCK	29	ROS1	67
CDK2	9	LIMK1	0	RPS6KA4 (Kin. Dom.1-N-term.)	26
CDK3	13	LIMK2	0	RPS6KA4 (Kin. Dom.2-C-term.)	89.3
CDK4	7	LKB1	0	RPS6KA5 (Kin. Dom.1-N-term.)	4
CDK4-cyclinD1	9	LOK	73	RPS6KA5 (Kin. Dom.2-C-term.)	89.4
CDK4-cyclinD3	22	LRRK2	**96**	RSK1 (Kin. Dom.1-N-term.)	0
CDK5	1	LRRK2 (G2019S)	89	RSK1 (Kin. Dom.2-C-term.)	60
CDK7	**95.8**	LTK	64	RSK2 (Kin. Dom.1-N-term.)	81
CDK9	0	LYN	9	RSK2 (Kin. Dom.2-C-term.)	0
CDKL1	42	LZK	0	RSK3 (Kin. Dom.1-N-term.)	2
CDKL2	13	MAK	5	RSK3 (Kin. Dom.2-C-term.)	74
CDKL3	7	MAP3K1	6	RSK4 (Kin. Dom.1-N-term.)	77
CDKL5	0	MAP3K2	79	RSK4 (Kin. Dom.2-C-term.)	36
CHEK1	2	MAP3K3	68	S6K1	51
CHEK2	48	MAP3K4	15	SGK	**91.1**
CIT	**92.8**	MAP4K2	53	SgK110	0
CLK1	85	MAP4K3	11	SGK2	88
CLK2	**95.9**	MAP4K4	47	SGK3	55
CLK3	19	MAP4K5	12	SIK	9
CLK4	**93.9**	MAPKAPK2	9	SIK2	7
CSF1R	0	MAPKAPK5	2	SLK	63
CSF1R-autoinhibited	37	MARK1	24	SNARK	90
CSK	21	MARK2	36	SNRK	22
CSNK1A1	**93.5**	MARK4	20	SRC	28
CSNK1A1L	63	MEK1	**93.1**	SRMS	29
CSNK1D	79	MEK2	87	SRPK1	53
CSNK1E	80	MEK3	**94**	SRPK3	66
CSNK1G1	84	MEK6	3	STK16	72
CSNK1G2	90	MELK	56	STK33	62
CSNK1G3	83	MERTK	4	STK35	1

CTK	0	MET	63	STK36	10
DAPK1	**96.3**	MET (M1250T)	51	STK39	53
DAPK2	**98.9**	MET (Y1235D)	25	SYK	20
DAPK3	**98.5**	MINK	70	TAK1	67
DCAMKL1	29	MKK7	0	TAOK1	62
DCAMKL2	0	MKNK1	72	TAOK2	37
DCAMKL3	10	MKNK2	**93.2**	TAOK3	65
DDR1	0	MLCK	11	TBK1	46
DDR2	0	MLK1	18	TEC	9
DLK	23	MLK2	4	TESK1	9
DMPK	49	MLK3	31	TGFBR2	24
DMPK2	3	MRCKA	17	TIE1	12
DRAK1	76	MRCKB	0	TIE2	21
DRAK2	78	MST1	32	TLK1	19
DYRK1A	**98.8**	MST1R	0	TLK2	0
DYRK1B	**96.4**	MST2	39	TNIK	65
DYRK2	81	MST3	62	TNK2	2
EGFR	15	MTOR	56	TRKA	0
EGFR (G719C)	24	MUSK	0	TRKB	34
EGFR (G719S)	13	MYLK	**93.9**	TRKC	31
EGFR (L747-T751 del, Sins)	22	MYLK2	30	TRPM6	8
EGFR (L858R)	17	MYLK4	7	TSSK1B	0
EGFR (L858R, T790M)	2	MYO3A	51	TSSK3	61
EGFR (L861Q)	12	MYO3B	30	TTK	**95.6**
EGFR (S752-I759 del)	16	NDR1	34	TYK2 (JH1 domain-cat.)	90
EGFR (T790M)	0	NDR2	13	TYK2 (JH2 domain-pseudokinase)	89.8
EIF2AK1	40	NEK1	31	TYRO3	18
EPHA2	19	NEK10	**100**	ULK1	15
EPHA3	45	NEK11	46	ULK2	52
EPHA5	9	NEK2	16	ULK3	55
EPHA7	9	NEK4	1	VEGFR2	39
EPHA8	0	NEK5	7	VPS34	27
EPHB1	0	NEK6	17	VRK2	0
EPHB2	11	NEK7	26	WEE1	16
EPHB3	0	NEK9	32	WEE2	0
EPHB4	0	NIM1	52	WNK1	12
EPHB6	80	NLK	11	WNK2	0
ERBB2	29	OSR1	25	WNK3	28
ERBB3	0	p38-α	0	WNK4	1
ERBB4	4	p38-β	15	YANK1	11
ERK1	0	p38-σ	7	YANK2	0
ERK2	8	PAK1	0	YANK3	0
ERK3	0	PAK2	41	YES	26
ERK4	0	PAK3	67	YSK1	10
ERK5	0	PAK4	43	MAP3K19	**95.8**
ERK8	50	PAK6	39	ZAK	14
ERN1	29	PAK7	55	ZAP70	2

*Abbreviations: P, phosphorylated form; non-P, non-phosphorylated form; cat., catalytic; term., terminal; kin., kinase. Residues are indicated in brackets for mutated forms. The percentage of binding inhibition for the 31 kinases for which >90% inhibition of ligand binding was observed is shown in bold.
